# THE DO IT MYSELF VILLAGE: BUILDING A VILLAGE-LIKE SUPPORT SYSTEM WITHOUT A VILLAGE

**DOI:** 10.1093/geroni/igz038.1522

**Published:** 2019-11-08

**Authors:** Allen Glicksman, Lauren Ring, Carrie Graham

**Affiliations:** 1 Philadelphia Corporation for Aging, Philadelphia, Pennsylvania, United States; 2 University of California Berkeley, Berkeley, California, United States

## Abstract

Villages provide members with a wide range of support including socialization, vetted vendors and other services that assist the elder to age in place. While not every Village offers the same types of support many older adults join Villages to gain benefits they may have lost (such as an informal support network) or ones they cannot find (such as identifying reliable providers of home repair). However, Villages are not available everywhere and there are barriers to Village membership, especially cost. Do older adults without access to a Village simply “do without” or do some of them create the same type of support system on their own? This presentation, using data collected in focus groups and individual interviews for a study of aging in community will describe the ways in which older adults have fashioned their own set of services and socialization opportunities to achieve the same goals as Village membership.

